# Obesity, change of body mass index and subsequent physical and mental health functioning: a 12-year follow-up study among ageing employees

**DOI:** 10.1186/s12889-017-4768-8

**Published:** 2017-09-26

**Authors:** Anna Svärd, Jouni Lahti, Eira Roos, Ossi Rahkonen, Eero Lahelma, Tea Lallukka, Minna Mänty

**Affiliations:** 1 Department of Public Health, Faculty of Medicine, University of Helsinki, (Tukholmankatu 8B), P.O. Box 20, 00014 Helsinki, Finland; 2 Finnish Institute of Occupational Health, Helsinki, Finland; 3 Laurea University of Applied Sciences, Unit of Research, Development and Innovation, Vantaa, Finland

**Keywords:** Body mass index, Obesity, Weight gain, Sf-36, Physical health functioning, Mental health functioning, Follow-up

## Abstract

**Background:**

Studies suggest an association between weight change and subsequent poor physical health functioning, whereas the association with mental health functioning is inconsistent. We aimed to examine whether obesity and change of body mass index among normal weight, overweight and obese women and men associate with changes in physical and mental health functioning.

**Methods:**

The Helsinki Health Study cohort includes Finnish municipal employees aged 40 to 60 in 2000–02 (phase 1, response rate 67%). Phase 2 mail survey (response rate 82%) took place in 2007 and phase 3 in 2012 (response rate 76%). This study included 5668 participants (82% women). Seven weight change categories were formed based on body mass index (BMI) (phase 1) and weight change (BMI change ≥5%) (phase 1–2). The Short Form 36 Health Survey (SF-36) measured physical and mental health functioning. The change in health functioning (phase 1–3) score was examined with repeated measures analyses. Covariates were age, sociodemographic factors, health behaviours, and somatic ill-health.

**Results:**

Weight gain was common among women (34%) and men (25%). Weight-gaining normal weight (−1.3 points), overweight (−1.3 points) and obese (−3.6 points) women showed a greater decline in physical component summary scores than weight-maintaining normal weight women. Among weight-maintainers, only obese (−1.8 points) women showed a greater decline than weight-maintaining normal weight women. The associations were similar, but statistically non-significant for obese men. No statistically significant differences in the change in mental health functioning occurred.

**Conclusion:**

Preventing weight gain likely helps maintaining good physical health functioning and work ability.

## Background

Obesity is a global threat to public health with the majority of adults in the OECD countries being overweight and one-fifth obese [1, 2]. In some countries the increase in obesity is slowing, but among middle-aged Europeans weight-gain remains common [3] and the worldwide prevalence [4] and the burden [5] attributable to obesity are still increasing. Obesity, but also weight gain itself, is associated with several public health issues, such as somatic diseases [6], work disability [7], premature mortality [8], poor quality of life and health functioning [9–18]. However, weight-loss has shown only small or no beneficial effects on health functioning [9–14]. Due to increased retirement age and life expectancy [19], the number of older employees is increasing. Health functioning is closely related to both work ability, quality of life, and ageing [20, 21], and therefore, factors associated with health functioning are important to study especially among ageing employees. In addition to prevention of obesity altogether, it is important for further studies to focus on factors that help maintaining good health, work ability and functioning in already-obese individuals.

Poor physical and mental health functioning, measured by the Short Form 36 Health Survey (SF-36), or similar other measures, are associated with obesity [15, 16, 22, 23]. A meta-analysis based on eight cross-sectional studies showed a dose—response association between body mass index (BMI) and poor physical health functioning [15]. The association with poor mental health functioning, however, occurred only for the obese with BMI ≥40 kg/m^2^. Also longitudinal studies suggest an association between obesity and poor subsequent physical, and possibly also mental health functioning [9–14, 17, 18].

Similarly to obesity, weight gain itself also associates with poor and declining physical health functioning, but inconsistently with mental health functioning [9–14, 17, 18]. A large study showed that weight gain among U.S. nurses aged 29 to 71 was associated with lower physical health functioning, but non-significantly with lower mental health functioning [9]. In contrast, greater weight-loss (>6,75 kg) among the overweight and obese participants was associated with better physical, but poorer mental health functioning. However, previous studies have shown inconsistent results and the effect of weight-loss is particularly poorly understood.

Comparing studies is challenging due to study-design heterogeneity; differences in follow-up time, measurements of weight change, and chose of statistical methods. In addition, some previous studies have been relatively small-scale [10–12, 17]. It seems that the associations with health functioning are stronger among women than men, but some large studies have only included women [9, 13].

Because obesity and weight-gain are associated with metabolic stress and inflammation [24, 25], consequently weight-gainers and those with the highest BMI could be at increased risk for obesity-related complications and poor health functioning. To judge whether weight-maintenance protects normal weight, overweight and obese employees from developing poor health functioning, longitudinal data are needed to examine the effect of weight change, in addition to body mass index, on physical and mental health functioning.

We aimed to deepen the understanding of the associations between BMI, weight change and health functioning by comparing the effect of weight change on physical and mental health functioning among different weight groups among midlife female and male employees. In addition, we adjusted for several covariates including age, marital status, socioeconomic position, employment status, smoking, drinking problem, physical activity, and somatic ill-health, as these factors are associated with health functioning and are often unequally distributed between the weight groups [26–28].

## Methods

### Data

The Helsinki Health Study cohort includes 8960 municipal employees of the City of Helsinki, Finland, aged 40 to 60 in 2000–02 (phase 1, response rate 67%). Follow-up mail surveys were conducted in 2007 (phase 2, response rate 82%) and in 2012 (phase 3, response rate 76%). Altogether 6245 participants responded to all three phases. Women were in the majority (82%), which reflects the gender distribution in the Finnish municipal sector. Men, younger employees and manual workers were slightly underrepresented, but according to non-response analyses, the data represent the target population satisfactorily [29, 30]. Pregnant (*n* = 23), underweight at phase 1 (BMI <18.5 kg/m^2^) (*n* = 53), and participants retired due to disability (*n* = 397) were excluded. The final data in analyses consisted of 4645 women and 1023 men after exclusions of responders with missing information on height or weight (*n* = 100), physical and mental health functioning at all phases 1–3 (*n* = 4). In the final data 21% of the participants had retired at phase 2 and 41% at phase 3.

The ethics committees of the Department of Public Health, University of Helsinki and the health authorities of the City of Helsinki approved the Helsinki Health Study protocol.

### Measures

#### Body mass index

The self-reported weight (kg) divided by the square of the height (m) defined the BMI (kg/m^2^), and a BMI change ≥5% between phase 1 and phase 2 defined the weight change. As long as the height is stable, a percentage change of BMI is equal with a percentage change in kilograms. Based on the weight change and BMI at phase 1, the participants formed seven groups: 1) normal weight weight-maintainers (BMI 18.5–24.9 kg/m^2^, weight change ≤5%), 2) normal weight weight-gainers (BMI 18.5–24.9 kg/m^2^, weight gain ≥5%), 3) overweight weight-maintainers (BMI 25–29.9 kg/m^2^, weight change ≤5%), 4) overweight weight-gainers (BMI 25–29.9 kg/m^2^, weight gain ≥5%), 5) obese weight-maintainers (BMI ≥30 kg/m^2^, weight change ≤5%), 6) obese weight-gainers (BMI ≥30 kg/m^2^, weight gain ≥5%) and 7) all weight-losers (weight loss ≥5%).

#### Physical and mental health functioning

The SF-36 focuses on self-assessed well-being and functioning and is a widely used measure of general health and quality of life [20]. The measure includes eight subscales scored from 0 to 100: physical functioning, role limitations due to physical health problems, bodily pain, general health perceptions, vitality, social functioning, role limitations due to emotional problems and mental health. The SF-36 physical component summary (PCS) and mental component summary (MCS) scores can be calculated from the subscales by means of factor analyses to measure general physical and mental health functioning. The component summary scores range from 0 to 100, with a mean of 50 and a standard deviation of 10 observed in the general US population. Higher scores indicate better functioning. A change greater than 3.0 points can be regarded as clinically significant [31].

#### Covariates

Phase 1 age included five categories: 40, 45, 50, 55 and 60. Socioeconomic position (SEP), measured at phase 1, consisted of managers and professionals, semi-professionals, routine non-manual employees, and manual workers [32]. Employment status categories at phase 2 and 3 were non-employed and employed, including part-time workers. Other covariates included measurements from all three phases and were used as time-variant. Marital status included married and cohabitants, and unmarried. Based on self-reported estimates of average weekly hours of leisure-time physical activity per each four intensity grades i.e. walking, brisk walking, jogging, running, or their equivalent activities, we calculated total leisure-time metabolic equivalent (MET) hours per week by multiplying the weekly hours by the MET value [33] of each physical activity intensity grade and adding the four values together [34]. Less than 14 MET-hours per week indicated physical inactivity (e.g. 2.5 h of brisk walking equals 15 MET-hours) [35]. Drinking problems as measured on the CAGE-questionnaire [36] included problem drinking and no problem drinking (the cut-off scores were two and three points for women and men, respectively). Smoking status included smokers, ex-smokers and non-smokers. Somatic ill-health was considered to be present among those who reported that a doctor had ever diagnosed them with at least one of the following diseases: gout, osteoarthrosis, rheumatoid arthritis, angina pectoris, myocardial infarction, claudication, epilepsy, or disturbance of the cerebral circulation. Participants non-reporting marital status, employment status, physical activity, problem drinking, smoking and somatic ill-health were considered as singles, employed, active, no problem drinkers, non-smokers, and healthy.

### Statistical analyses

**Fig. 1:**
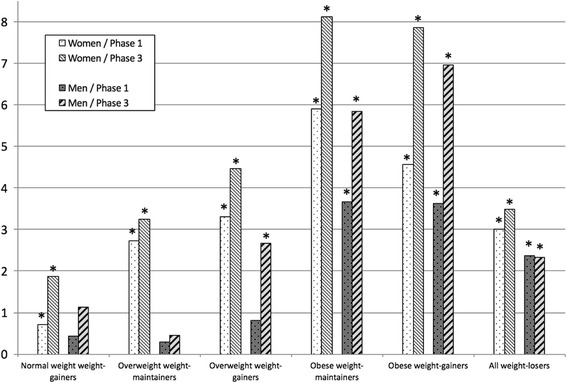
Age-adjusted mean differences of SF-36 physical component summary (PCS) score at phase 1 and phase 3 compared with normal weight weight-maintaining women (*n* = 4407) and men (*n* = 976); * *p* < 0.05

**Fig. 2:**
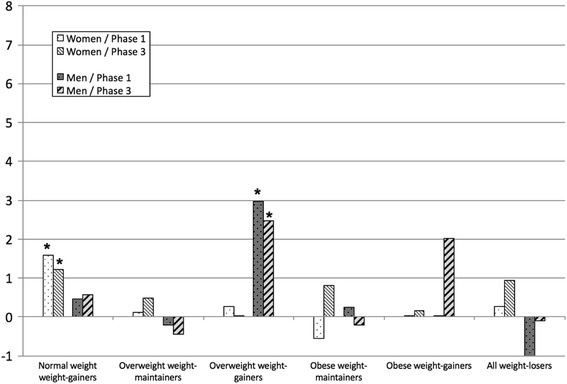
Age-adjusted mean differences of SF-36 mental component summary (MCS) score at phase 1 and phase 3 compared with normal weight weight-maintain maintaining women (*n* = 4407) and men (*n* = 976); * p < 0.05

Firstly, we used cross-tabulation to describe the phase 1 characteristics (Table 1). Secondly, we calculated the mean scores and standard deviations for the physical and mental health functioning by weight change groups at all three phases (Table 2). Thirdly, we calculated adjusted cross-sectional differences in PCS and MCS scores at phases 1 and 3 by weight change groups using linear regression analysis (Figs. 1 and 2). Fourthly, we calculated adjusted differences in the changes of PCS and MCS scores from phase 1 to phase 3 (including phase 2) with repeated measures analysis using MIXED procedure in SPSS (Tables 3 and 4). The results are reported as regression coefficients (β) and their standard errors (SE). Normal weight weight-maintainers served as a reference group. Model 1 adjusted for age, and model 2, model 3, and model 4 adjusted additionally for socio-demographic factors, health behaviours and somatic ill-health, respectively. All covariates, except for socioeconomic position, functioned as time variants. Women and men were examined separately in all analyses due to a gender interaction (PCS *p* = 0.008; MCS *p* = 0.006). We conducted the analyses with IBM SPSS Statistics 23.

**Table 1:** Percentage of women and men by phase 1 characteristics and mean SF-36 physical component summary (PCS) and mental component summary (MCS) scores at phase 1

	Women			Men		
Charasteristics at Phase 1	%	Mean PCS^a^	Mean MCS^b^	%	Mean PCS^a^	Mean MCS^b^
*Age (years)*						
40	19	51.1	51.8	15	51.8	50.4
45	21	50.9	51.5	18	52.6	51.0
50	21	49.8	52.2	21	50.6	50.8
55	25	48.1	51.9	30	50.4	52.4
60	13	46.5	52.2	16	49.3	53.9
*BMI* ^*c*^						
Normal weight	56	51.1	51.7	42	52.0	51.8
Overweight	31	47.9	52.0	45	50.9	51.7
Obese	13	45.8	52.4	13	47.3	52.0
*Weight change* ^*d*^						
Weight maintenance	56	49.7	52.2	62	51.2	52.1
Weight gain	33	49.6	51.3	24	50.9	50.3
Weight loss	11	47.7	52.3	14	49.1	53.1
*Marital status*						
Married and cohabiting	68	49.5	52.3	80	50.8	52.2
Unmarried	32	49.3	51.0	20	50.9	50.1
*Socioeconomic position*						
Managers/professionals	29	50.7	50.8	48	51.9	51.4
Semi-professionals	21	50.3	51.5	20	50.8	52.2
Routine non-manual	38	48.6	52.7	8	49.6	51.7
Manual workers	13	47.7	52.7	24	49.2	52.3
*Smoking*						
Non-smoker	20	50.0	51.0	23	49.9	49.9
Ex-smoker	58	49.3	52.2	45	51.3	52.2
Smoker	22	49.7	52.0	33	50.8	52.6
Drinking problems (CAGE)						
No problem drinking	85	49.4	52.6	77	51.0	52.7
Drinking problem	15	49.6	47.8	23	50.4	48.7
*Physical activity (MET)*						
Active	77	50.0	52.2	76	51.6	52.2
Inactive	23	47.6	50.7	24	48.4	50.7
*Somatic ill-health*						
No somatic disease	79	50.7	51.9	79	51.7	51.9
Somatic disease	21	44.7	51.8	21	47.6	51.3
*Total*	4524	49.4	51.9	1000	50.8	51.8

^a^ Physical component summary score (PCS) derived from Short Form 36 Health Survey (SF-36)

^b^ Mental component summary score (MCS) derived from Short Form 36 Health Survey (SF-36)

^c^ Body mass index (BMI): normal weight 18.5–24.9 kg/m^2^, overweight 25–29.9 kg/m^2^, and obese ≥30 kg/m^2^

^d^ BMI change ≥5% (Phase 1–2)

**Table 2:** Mean physical component summary (PCS) and mental component summary (MCS) scores and their standard deviation (SD) at phase 1, phase 2, and phase 3 by weight change groups

		Mean PCS score^a^ (SD)			Mean MCS score^b^ (SD)		
	BMI^c^ (change ≥5%)	Phase 1	Phase 2	Phase 3	Phase 1	Phase 2	Phase 3
Women	Normal weight						
	Stable weight	51.3 (7.0)	50.4 (7.6)	49.6 (7.9)	52.2 (8.9)	52.7 (9.3)	53.6 (8.6)
	Weight gain	51.0 (7.5)	48.6 (8.1)	47.9 (8.9)	50.7 (10.0)	51.9 (9.3)	52.2 (9.6)
	Overweight						
	Stable weight	48.0 (8.2)	47.4 (8.6)	46.1 (9.2)	52.1 (9.4)	52.8 (9.4)	53.4 (8.9)
	Weight gain	48.2 (8.1)	46.6 (8.7)	45.2 (9.3)	52.0 (9.6)	52.5 (9.8)	53.6 (8.8)
	Obese						
	Stable weight	45.0 (9.2)	43.0 (10.4)	41.4 (9.5)	52.2 (9.5)	53.2 (9.7)	53.0 (10.2)
	Weight gain	46.8 (8.2)	42.9 (9.5)	41.6 (9.9)	52.2 (10.6)	52.4 (9.3)	53.3 (10.3)
	All						
	Weight loss	47.7 (8.6)	47.5 (9.4)	45.9 (9.7)	52.3 (9.5)	51.8 (10.7)	53.1 (9.7)
	Total	49.4 (8.0)	48.1 (8.7)	47.1 (9.2)	51.9 (9.5)	52.5 (9.6)	53.2 (9.2)
		*n* = 4524	*n* = 4524	*n* = 4513	*n* = 4524	*n* = 4524	*n* = 4513
Men	Normal weight						
	Stable weight	52.1 (6.3)	51.4 (6.5)	50.6 (7.2)	52.0 (9.6)	52.8 (8.2)	52.9 (9.0)
	Weight gain	51.9 (6.2)	51.1 (6.6)	49.8 (7.4)	51.1 (9.5)	52.3 (9.2)	51.8 (10.5)
	Overweight						
	Stable weight	51.2 (6.3)	50.1 (7.2)	49.8 (7.4)	52.2 (8.6)	52.3 (9.7)	53.6 (8.5)
	Weight gain	50.9 (6.8)	48.8 (7.4)	48.3 (8.7)	48.8 (11.0)	50.2 (10.0)	49.9 (11.1)
	Obese						
	Stable weight	47.4 (8.1)	45.7 (8.7)	44.6 (9.6)	52.0 (9.5)	53.8 (8.3)	53.7 (8.5)
	Weight gain	47.9 (8.3)	43.9 (9.4)	43.3 (10.2)	52.3 (9.1)	53.1 (9.5)	51.3 (12.7)
	All						
	Weight loss	49.1 (8.0)	59.4 (7.2)	48.0 (8.2)	53.1 (9.2)	54.2 (8.9)	53.4 (9.9)
	Total	50.8 (7.0)	49.8 (7.4)	49.0 (8.1)	51.8 (9.5)	52.6 (9.1)	52.7 (9.5)
		*n* = 1000	*n* = 1007	*n* = 999	*n* = 1000	*n* = 1007	*n* = 999

^*a*^ *Physical component summary score (PCS) derived from Short Form 36 Health Survey (SF-36)*

^*b*^ *Mental component summary score (MCS) derived from Short Form 36 Health Survey (SF-36)*

^*c*^ *Body mass index (BMI): normal weight 18.5–24.9 kg/m* ^*2*^ *, overweight 25–29.9 kg/m* ^*2*^ *, and obese ≥ 30–34.9 kg/m* ^*2*^

**Table 3:** Adjusted mean differences in changes in Physical Health Functioning Score (PCS) between phase 1 and phase 3 by different weight change categories

			Model 1		Model 2		Model 3		Model 4	
			Phase 1- > Phase 3		Phase 1- > Phase 3		Phase 1- > Phase 3		Phase 1- > Phase 3	
BMI (change ≥5%)^*a,b*^		n (%)	β	SE	β	SE	β	SE	β	SE
Women	Normal weight									
	Stable weight^c^	1538 (33)	0	0	0	0	0	0	0	0
	Weight gain	893 (19)	−1.3*	0.4	−1.2*	0.4	−1.2*	0.4	−1.2*	0.4
	Overweight									
	Stable weight	786 (17)	−0.2	0.4	−0.3	0.4	−0.1	0.5	−0.3	0.5
	Weight gain	470 (10)	−1.3*	0.5	−1.2*	0.5	−1.0	0.6	−1.2*	0.6
	Obese									
	Stable weight	270 (6)	−1.8*	0.7	−1.9*	0.7	−1.6*	0.7	−1.7*	0.7
	Weight gain	188 (4)	−3.6*	0.8	−3.7*	0.8	−3.2*	0.8	−3.3*	0.8
	All									
	Weight loss	500 (11)	0.0	0.5	−0.2	0.5	0.1	0.6	0.4	0.5
Men	Normal weight									
	Stable weight^c^	293 (29)	0	0	0	0	0	0	0	0
	Weight gain	102 (10)	−0.5	1.0	−0.5	1.0	−0.6	1.0	−1.2	1.0
	Overweight									
	Stable weight	277 (27)	0.1	0.7	0.1	0.7	0.1	0.7	−0.4	0.7
	Weight gain	107 (11)	−1.2	1.0	−1.2	1.0	−1.2	1.0	−1.4	1.0
	Obese									
	Stable weight	68 (7)	−1.6	1.2	−1.7	1.2	−1.9	1.2	−1.7	1.1
	Weight gain	36 (4)	−3.5*	1.5	−3.6*	1.5	−3.4*	1.5	−3.5*	1.5
	All									
	Weight loss	140 (14)	0.6	0.9	0.5	0.9	0.3	0.9	−0.1	0.9

*Model 1: Adjusted for age*

*Model 2: Adjusted for age and socio-demographic factors (marital status, socioeconomic position, and employment status)*

*Model 3: Adjusted for age and health behaviours (smoking, problem drinking and physical activity)*

*Model 4: Adjusted for age and somatic ill-health*

*p < 0.05

^*a*^ *Body mass index (BMI): normal weight 18.5–24.9 kg/m* ^*2*^ *, overweight 25–29.9 kg/m* ^*2*^ *, and obese ≥ 30–34.9 kg/m* ^*2*^

^*b*^ *BMI change ≥ 5% (Phase 1–2)*

^*c*^ *Reference group*

**Table 4:** Adjusted mean differences in changes in Mental Health Functioning Score (MCS) between phase 1 and phase 3 by different weight change categories

			Model 1		Model 2		Model 3		Model 4	
			Phase 1- > Phase 3		Phase 1- > Phase 3		Phase 1- > Phase 3		Phase 1- > Phase 3	
BMI (change ≥5%)^*a,b*^		n^d^ (%)	β	SE	β	SE	β	SE	β	SE
Women	Normal weight									
	Stable weight^c^	1538 (33)	0	0	0	0	0	0	0	0
	Weight gain	893 (19)	0.3	0.5	0.3	0.5	0.4	0.5	0.3	0.5
	Overweight									
	Stable weight	786 (17)	−0.1	0.5	−0.2	0.5	−0.2	0.5	−0.1	0.5
	Weight gain	470 (10)	0.1	0.6	0.1	0.6	0.1	0.6	0.9	0.6
	Obese									
	Stable weight	270 (6)	−0.5	0.8	−0.7	0.8	−0.6	0.8	−0.5	0.8
	Weight gain	188 (4)	−0.2	0.9	−0.2	0.9	−0.1	0.9	−0.2	0.9
	All									
	Weight loss	500 (11)	−0.7*	0.6	−0.8	0.6	−0.6	0.6	−0.7	0.6
Men	Normal weight									
	Stable weight^c^	293 (29)	0	0	0	0	0	0	0	0
	Weight gain	102 (10)	−0.1	1.3	0.0	1.3	−0.2	1.3	−0.3	1.3
	Overweight									
	Stable weight	277 (27)	0.4	0.9	0.3	0.9	0.4	0.9	0.4	0.9
	Weight gain	107 (11)	0.3	1.3	0.5	1.3	0.2	1.3	0.2	1.3
	Obese									
	Stable weight	68 (7)	1.0	1.5	0.9	1.5	0.4	1.5	0.9	1.5
	Weight gain	36 (4)	−2.0	2.0	−2.1	2.0	−2.1	2.0	−2.0	2.0
	All									
	Weight loss	140 (14)	−0.5	1.2	−0.5	1.1	−0.6	1.1	−0.6	1.2

*Model 1: Adjusted for age*

*Model 2: Adjusted for age and socio-demographic factors (marital status, socioeconomic position, and employment status)*

*Model 3: Adjusted for age and health behaviours (smoking, problem drinking and physical activity)*

*Model 4: Adjusted for age and somatic ill-health*

*p < 0.05

^*a*^ *Body mass index (BMI): normal weight 18.5–24.9 kg/m* ^*2*^ *, overweight 25–29.9 kg/m* ^*2*^ *, and obese ≥ 30–34.9 kg/m* ^*2*^

^*b*^ *BMI change ≥5% (Phase 1–2)*

^*c*^ *Reference group*

## Results

At phase 1 13% of both women and men were obese, whereas 45% of men and 31% of women were overweight (Table 1). Between phase 1 and phase 2 weight gain was common among women (33%) and men (24%), whereas weight-loss occurred less often (women 11%, men 14%). Phase 1 PCS scores were low among those with high age, obesity, low SEP, physical inactivity and somatic ill-health at phase 1, whereas MCS scores were low among those with drinking problems. The mean PCS scores at phase 1 tended to be higher among men than women.

The mean PCS scores showed an inverse dose-response association with BMI, showing that PCS decreased with increasing BMI, whereas for MCS there was no such association (Table 2). Regression analyses showed that compared to normal weight weight-maintainers the age-adjusted differences in PCS scores between the weight groups were statistically significant for the overweight and obese women at phase 1 and phase 3 (Fig. 1). Among men the differences in PCS scores were significant for the obese at phase 1 and 3 and for the overweight weight-gainers at phase 3. Among weight-losers both women and men showed a lower PCS score than the normal weight weight-maintainers (Fig. 1). The mean MCS scores were similar in the weight groups (Table 2). However, the regression analyses showed that weight-gaining normal weight women and weight-gaining overweight men had lower MCS scores than the normal weight weight-maintainers at phase 1 (Fig. 2).

In addition to the dose—response association found for PCS, the mean scores also suggested a time trend for both PCS and MCS. However, in contrast to PCS, MCS mean scores showed a slight increase over time (Table 2). When the differences in the change of PCS mean scores were examined with repeated measures analyses, weight gain among women was associated with a greater decline, irrespective of phase 1 BMI: weight-gaining normal weight (−1.3 points), overweight (−1.3 points) and obese (−3.6 points) women showed a greater decline in PCS scores than weight-maintaining normal weight women (Table 3). Among weight-maintainers, only obese (−1.8 points) women showed a greater decline.

Adjustment for health behaviours and somatic ill-health slightly attenuated the associations, and except for the weight-gaining overweight women, the changes remained statistically significant (Table 3). There was a similar, but weaker, difference between weight-gaining overweight, weight-gaining obese, and weight-maintaining obese men. Only for the weight-gaining obese men, the decrease was statistically significant, even after adjustments. Among weight-losing women and men there were no significant changes in PCS scores.

In line with the mean scores, the analyses showed no statistically significant differences in the changes in MCS scores for weight-maintainers and weight-gainers (Table 4). Weight-losing women showed a − 0.7 points difference to the reference group, and adjustment for covariates slightly attenuated the estimate. Weight-gaining obese men showed a non-significant difference in MCS score (−2.0 points).

## Discussion

### Principal findings

Obese but also weight-gaining women showed worse and declining physical health functioning compared with normal weight weight-maintaining women. These findings were similar, but statistically weaker for obese men. The changes in mental health functioning, however, did not differ between the weight groups. Adjusting for health behavior and somatic ill-health showed small, but mainly statistically non-significant effects, on the findings. Weight-loss did not show a positive association with health functioning.

### Comparison to previous studies

This study confirms the findings from others suggesting that physical health functioning declines faster among the obese. As expected, also weight gain was associated with a greater decline in physical health functioning. Obesity and weight gain increase musculoskeletal strain, but may also add to the internal metabolic stress and inflammation [24, 25]. Therefore, it is possible that besides obesity, weight-gain itself also contributes to the genesis of inflammation-mediated physical and mental conditions (e.g. depression, diabetes, and cardiovascular diseases) [37, 38], which might explain why weight-gaining women show a greater decline in physical health functioning than weight-maintaining women. However, no association occurred for mental health functioning. It is possible that the decline in physical, and especially mental health functioning is too slow to be observed within a decade of follow-up. To reconfirm our findings, further studies with longer and more frequent follow-ups within different cohorts are needed.

As in previous studies, the association between weight change and mental health functioning remained less clear also in our study. A meta-analysis showed an association with mental health functioning only for the morbidly obese with BMI > 40 kg/m^2^ [15]. In our study the morbidly obese (BMI ≥35 kg/m^2^) women (*n* = 136) and men (*n* = 30) were few. We however, examined the severely obese separately in sensitivity analyses. Weight-gaining and weight-losing severely obese women showed a greater decline in PCS scores than the weight-gaining and weight-losing obese with BMI 30–35 kg/m^2^. Among the severely obese no associations were found for MCS.

In our study, among weight-losing women a weak association with a greater decline in mental health functioning occurred, whereas there was no association for physical health functioning. However, for reliable analysis of the weight-losers intended and unintended weight-loss should be distinguished. We did adjust for self-reported somatic ill-health as time variant, but for reliable examination, unintended weight-loss due to somatic health problems, such as cancer, should be ruled out. Intended weight-loss among the obese individuals would improve especially physical health functioning according to findings from a clinical trial [39]. In addition, also when examining mental health functioning, it is important to distinguish between intended and unintended weight loss, as depression as well as other mental problems might cause unintended weight-changes and thus bias the results.

Weight-loss among women and weight-gain among obese men was associated with a non-significantly greater decline in mental health functioning. Common mental disorders such as anxiety disorder and psychotic disorders such as schizophrenia are associated with weight gain, which may possibly explain the association among the weight-gaining obese men [40, 41].

### Strengths and limitations

The strengths of this study include a large cohort including both women and men representing hundreds of different occupations. Three identical repeated measurements of the widely used SF-36 health functioning were available, which allowed the examination of the differences in changes over a decade of follow-up. Furthermore, several covariates were included at all three time points.

The study limitations include, firstly, that the data covered only middle-aged municipal employees. However, this growing population group is important to study because health functioning is closely related to work ability, quality of life and ageing [19–21]. Secondly, height and weight as well as covariates were based on self-reports. In these data self-reported BMI predicted sickness absence as accurately as did measured BMI, however [42]. Thirdly, obese (*n* = 84) and weight-losing (*n* = 115) men were few in number, which complicates reliable analyses for these groups. Fourthly, we were unable to distinguish between intended and unintended weight-loss. Many somatic diseases may cause unintended weight-loss, which might bias the results. However, we were able to adjust for a range of somatic diseases. Fifthly, the time between follow-ups was relatively long (5–7 years) with some unobserved changes in the data possibly taking place. Finally, the survey response rate was acceptable, but non-response and selection of healthy workers remains a problem. Healthy workers are likely overrepresented, which weakens the generalizability of the results [43]. The excluded participants who retired due to disability retirement had lower SF-36 scores at phase 1.

Also retirees that retired due to non-medical reasons showed lower SF-36 scores than the employed, however visually, the curves among the weight groups among the retired and the employed were similar. The interaction between these groups was statistically significant, but we considered the interaction to be of removable type [44], after examining retirees separately in sensitivity analyses and finding that female retirees at phase 2 showed a greater but similar decline in PCS and that including this group in the final analysis did not affect the final estimates or the conclusions.

## Conclusions

Besides obesity also weight gain associates with worse physical, but not mental health functioning. Weight-loss showed no positive association with either physical or mental health functioning. Preventing further weight gain may be beneficial in maintaining physical health functioning and work ability particularly among already-obese employees.

## Abbreviations

BMI: Body mass index
CI: Confidence interval
MET: Metabolic equivalent task
SEP: Socioeconomic position
SF-36: Short Form 36 Health Survey
WHO: World Health Organization
