# Correction to: Obesity, change of body mass index and subsequent physical and mental health functioning: a 12-year follow-up study among ageing employees

**DOI:** 10.1186/s12889-017-4828-0

**Published:** 2017-10-17

**Authors:** Anna Svärd, Jouni Lahti, Eira Roos, Ossi Rahkonen, Eero Lahelma, Tea Lallukka, Minna Mänty

**Affiliations:** 1 Department of Public Health, Faculty of Medicine, University of Helsinki, (Tukholmankatu 8B), P.O. Box 20, 00014 Helsinki, Finland; 2 Finnish Institute of Occupational Health, Helsinki, Finland; 3 Laurea University of Applied Sciences, Unit of Research, Development and Innovation, Vantaa, Finland

## Correction

After publication of the article [1], it has been brought to our attention that obesity has been incorrectly defined in the parameters below Tables 2, 3 and 4. As already in Table 1, it should be defined as ≥30 kg/m2. The original version of the article has been revised to reflect this.
